# An in-depth analysis of the molecular changes induced by short-term calorie restriction before living kidney donation

**DOI:** 10.1038/s41514-026-00401-w

**Published:** 2026-05-28

**Authors:** Martin R. Späth, Sita Arjune, Katrin Bohl, Markus M. Rinschen, Christine S. Falk, Philipp Antczak, Franziska Grundmann, Torsten Kubacki, K. Johanna R. Hoyer-Allo, Michael Ignarski, Felix C. Koehler, Kathrin Kaufmann, Malte P. Bartram, Tristan Wagner, Michael Thomas, Jan Wilm Lackmann, Adam Wahida, Christoph Schmaderer, Stephan Kemmner, Christine E. Kurschat, Thomas Benzing, Dirk Stippel, Volker Burst, Roman-Ulrich Müller

**Affiliations:** 1 Department II of Internal Medicine and Center for Molecular Medicine Cologne, University of Cologne, Faculty of Medicine and University Hospital Cologne, Cologne, Germany; 2 CECAD, University of Cologne, Faculty of Medicine and University Hospital Cologne, Cologne, Germany; 3 Department of Nephrology, Medical Faculty, University Hospital Düsseldorf, Heinrich-Heine-University Düsseldorf, Düsseldorf, Germany; 4 Policlinic for Endocrinology, Diabetes and Preventive Medicine (PEPD), University of Cologne, Faculty of Medicine and University Hospital Cologne, Cologne, Germany; 5 III. Department of Medicine, Hamburg Center for Kidney Health, University Medical Center Hamburg-Eppendorf, Hamburg, Germany; 6 Scripps Center for Metabolomics and Mass Spectrometry, The Scripps Research Institute, 10550 North Torrey Pines Road, California 92037 La Jolla, USA; 7 Department of Biomedicine, Aarhus University, Aarhus, Denmark; 8 Institute of Transplant Immunology, Hannover Medical School, Hannover, Germany; 9 Einthoven Laboratory of Vascular and Regenerative Medicine, Leiden University Medical Center, Leiden, the Netherlands; 10 Department of General, Visceral, Thoracic and Transplantation Surgery, University Hospital of Cologne, Cologne, Germany; 11 CECAD, University of Cologne, Faculty of Mathematics and Natural Sciences, Cologne, Germany; 12 Medical Department III of Hematology and Oncology, Klinikum rechts der Isar, TUM School of Medicine, Technical University of Munich, Munich, Germany; 13 Krantz Family Center for Cancer Research, Massachusetts General Hospital Cancer Center, Massachusetts Charlestown, USA; 14 Department of Molecular Metabolism, Harvard T.H. Chan School of Public Health, Massachusetts Boston, USA; 15 Clinical Division of Oncology, Department of Internal Medicine, Medical University of Graz, Graz, Austria; 16 Department of Nephrology, Klinikum rechts der Isar, TUM School of Medicine, Technical University of Munich, Munich, Germany; 17 Transplant Center, University Hospital Munich, Ludwig-Maximilians-University (LMU), Munich, Germany; 18 Emergency Department, University Hospital Cologne, Cologne, Germany; 19 Center for Rare Diseases Cologne, Faculty of Medicine and University Hospital Cologne, University of Cologne, Cologne, Germany

**Keywords:** Diseases, Medical research, Nephrology

## Abstract

Aging reduces cellular resilience and increases susceptibility to organ injury, notably acute kidney injury (AKI). Ischemia-reperfusion injury (IRI) influences outcomes after kidney transplantation. In animal models, short-term calorie restriction (CR) extends lifespan and protects kidneys from IRI, but translation to patients is limited due to incomplete mechanistic insight. This study examined clinical and molecular effects of short-term CR in living kidney donors. Twelve donors were alternately assigned to CR or an ad libitum diet. CR participants consumed a formula diet providing 50% of individual caloric needs for seven days before donation. Clinical parameters and biosamples from perirenal fat, renal arteries, ureters, kidney biopsies, blood, and urine were collected. Primary outcomes were CR-induced molecular changes; clinical outcomes were secondary. CR was well tolerated and caused significant weight loss without affecting AKI incidence or increasing adverse events. Lipidomic and proteomic analyses showed enhanced lipolysis and proteostasis, reduced insulin signaling, sex-specific effects, and decreased inflammatory factors in donor arteries and ureter tissue. This hypothesis-generating study indicates that short-term CR before donation is feasible and suggests that CR promotes organ protection in humans by dampening insulin signaling and inflammation, providing a basis for future targeted interventions in clinical and transplant research moving forward.

## Introduction

Chronic kidney disease (CKD) shows an increasing prevalence in aging societies during the last decades and in many cases results in a need for kidney replacement therapy^1^. In regard to mortality, quality of life and costs, kidney transplantation is superior to dialysis^2^. Despite several improvement attempts the organ demand markedly exceeds the organ supply^3^. Therefore, best possible conditions should be aimed for in each kidney transplantation. Ischemia-reperfusion injury (IRI) is a well-known factor promoting delayed graft function (DGF) shown to be associated with an earlier loss of organ function and mortality after transplant^4^. Thus, it is well-established to reduce ischemia-time to a minimum, and all possible resources should be used to reduce IRI-induced transplant impairment.

In several animal models, short-term calorie restriction (CR) has been demonstrated to positively impact on longevity across species^5–7^ and has also proven to reduce IRI-induced organ damage effectively. Despite several attempts, translation to the clinical setting lags behind, and a robust understanding of the underlying molecular mechanisms is still needed to achieve this step. In animal models, there is increasing evidence, that CR-induced protective effects are mediated due to an influence on ribosomal stabilization, insulin signaling, H_2_S- and NAD-metabolism as well as on inflammatory pathways^8–12^, but robust data in humans remain scarce.

Difficulties can be easily found in the higher heterogeneity due to sex, environment, body weight, co-morbidities and medication etc. of a human population in comparison to the homogeneous animal studies. Healthy living kidney donors are highly motivated and suited as ideal candidates for clinical pilot trials. Van Ginhoven and Jongbloed et al. had previously shown, that a short-term preoperative dietary restriction is feasible and may improve outcomes in healthy kidney donors^13–15^.

This hypothesis generating pilot-study investigates the molecular effects of a short-term CR in healthy living donors in a multiomics approach as a primary endpoint. Proteome profiles of kidney biopsies and lipidome data on perirenal adipose tissue samples were obtained. Besides, we performed proteomics and cytokine/chemokine analyses on longitudinally acquired blood serum samples as well as cross-sectional cytokine/chemokine analyses of renal artery and ureter specimens. The clinical outcome of the recipients was examined as a secondary endpoint.

## Results

### Baseline characteristics and clinical outcome

**Fig. 1:**
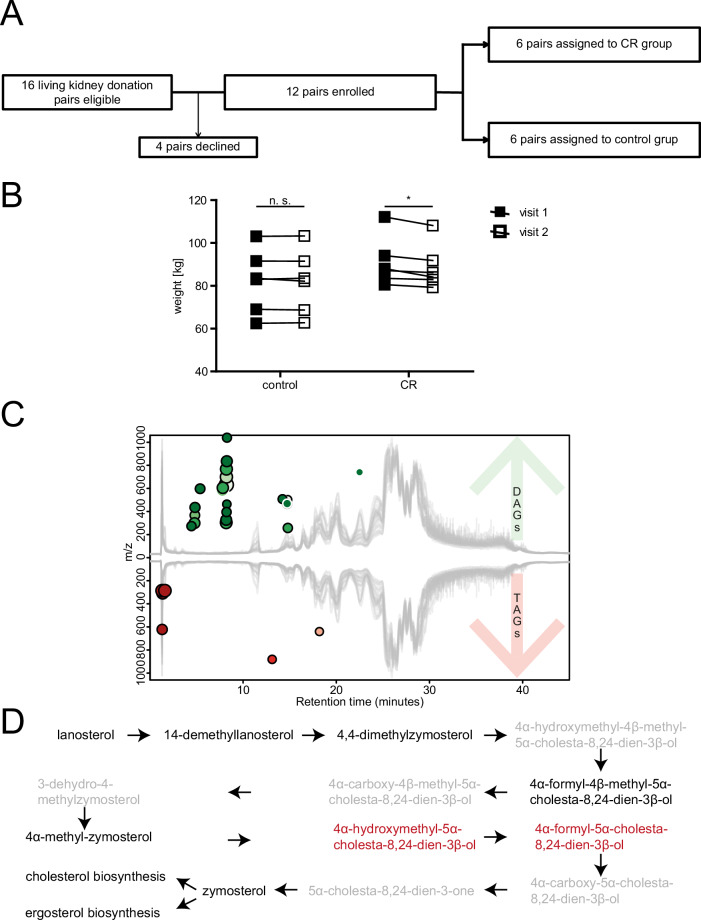
CONSORT flow diagram, effect on weight changes and lipidomics. **A** CONSORT Flow diagram. **B** Weight changes between visit 1 and 2 in control and CR group. **C** Cloud plot showing 34 features with positive polarization (*p*-value < 0.001, log2FC ≥ 2). CR caloric restriction, DAGs diacylglycerides, TAGs triacylglycerides. Green: upregulated; red: downregulated. **D** Zymosterol biosynthesis pathway (PWY-6074) modified from MetaCyc.org. black: compounds belonging to the biosynthesis pathway but not annotated to pathway PWY-6074; gray: compounds annotated to PWY-6074, red: compound indicating an enrichment of the pathway (*p* < 0.1).

The first patient was included on June 25, 2015, and the last follow up visit was scheduled on September 12, 2016. During this time, 16 kidney donor-recipient pairs were found eligible and were informed about the trial during clinical routine visits. Four donors declined their participation and following the study protocol, 12 pairs were screened, enrolled and alternately assigned to the intervention or control group (Fig. 1A). CR was calculated up to 50% of the DEE and CR had to be maintained until the evening of day −1 (for timeline see Supplementary Fig. S1A). All enrolled patients were included in the clinical analyses. The mean age in both groups was 52 years. In the control group 50% and in the CR-group 60% of patients were male. The mean BMI was 28 kg/m² in both groups at the first visit (Supplementary Fig. S1B) and the mean creatinine values at baseline, i.e. at the first consultation for the planning of the transplantation, were 0.97 mg/dL in the CR-Group and 0.82 mg/dL in the control group (Supplementary Fig. S1C). There were no significant differences between groups concerning age, sex, BMI, kidney function, surgical technique, length of the operation or AB0-status. Detailed patients’ demographics and clinical preoperative data are shown in Table 1.

**Table 1:** Patient demographics and clinical characteristics

	Donors			Recipients		
Parameter	CR	Control	*p*	CR	Control	*p*
Age (y), median (QR)	52.58 (11.2)	55.85 (11.7)	0.92	54.7 (21.04)	45.47 (27.66)	0.99
Male, *n* (%)	4 (60)	3 (50)	0.599	3 (50)	3 (50)	1
Height at screening [cm], median (QR)	177.5 (10)	171 (20)	0.2	170 (17)	172 (22)	0.896
Weight at screening [kg], median (QR)	87.6 (15.9)	83.3 (23.8)	0.3	85.5 (26.3)	78.5 (33.1)	0.397
BMI at screening [kg/m²], median (QR)	28 (6.6)	29.9 (8.8)	0.994	26.9 (5.8)	25.6 (4.4)	0.331
Creatinine pre-op, median [mg/dL]	1.01 (0.31)	0.87 (0.17)	0.171	7.43	5.41	
Creatinine eGFR pre-op without dialysis [ml/min]	87.5 (30)	79.5 (25.75)	0.442	6	10	
Cystatin-C pre-op, median [mg/L]	1.05 (0.33)	0.89 (0.11)	0.2	–	–	–
Cystatin-C eGFR pre-op without dialysis [ml/min]	78.5 (41)	96 (17)	0.18	–	–	–
Dialysis pre-op, *n* (%)	0	0	–	3 (50)	5 (83.33)	0.26
Urinary NGAL pre-op [ng/mL], mean (SD)	14.18 (8.53)	15 (14.21)	0.906	176.03 (260.25)	343.7	0.633
HARP, *n* (%)	6 (100)	6 (100)	1	–	–	–
Right nephrectomy, n(%)	3 (50)	3 (50)	1	–	–	–
AB0i, *n* (%)	2 (33.33)	2 (33.33)	1	–	–	–

*y* years, *QR* interquartile range, *SD* standard deviation.

The median daily calorie intake during the intervention as reported via diary entries was 1144.0 kcal (IQR 534.0) in the CR group and 1781.5 kcal (IQR 1171.8) in the control group representing a relative calorie intake of 49.99% in the CR group and 75.93% in the control group for the calculated daily energy expenditure (DEE). Despite the trend towards harder stools without clinical signs of evident obstipation, patients of the CR-group did not report any adverse effects of the interventions. All patients of the control group reported, that they did not change their dietary habits. The median weight difference between the first and second visit was −1.8 kg (−2.02%) in the CR group and 0.05 kg (0.04%) in the control group (*p* = 0.013 for absolute changes, 0.011 for relative changes) (Fig. 1B). As expected, the outcomes of all living kidney donations were very good. All nephrectomies were performed in the HARP-technique^16^, the median warm-ischemia time was 27 min in the CR group and 31 min in the control group (*p*: 0.67). No delayed graft function was detected and only one patient developed an acute kidney injury KDIGO I – i.e. creatinine increase ≥0.3 within 24 h – due to cellular rejection. Furthermore, CR did not show any effect on the change of NGAL or cystatin C, the maximum creatinine until discharge or the length of the hospital stay. Detailed data on the clinical outcome are shown in Table 2.

**Table 2:** Anthropometric, clinical and biochemical outcome characteristics

	Donors			Recipients		
Parameter	CR	Control group	*p*	After donor’s CR	Without CR	*p*
Delta weight: screening to day −1, median (QR) [kg]	−1.8 (3.03)	0.05 (1.22)	0.013	–	–	–
Delta relative weight: screening to day −1, median (QR) [%]	−2.02 (2.74)	0.04 (1.69)	0.011	–	–	–
Delta body water: screening to day −1, median Q1–Q3 [kg]	−2.15 (2.7)	−2.05 (2.68)	0.733	–	–	–
Calculated daily energy expenditure (DEE), median (QR) [kcal]	2419.11 (475)	2377.42 (924)	0.883	–	–	–
Reported caloric intake d1–d7, median QR [kcal]	1144 (534)	1781 (1171.68)	0.075	–	–	–
Reported caloric intake (d1–d7) in relation to DEE, median QR [%]	49.99 (21.82)	75.93 (43.68)	0.04	–	–	–
Nephrectomy of one own kidney	–	–	–	1 (16.67)	1 (16.67)	1
Cold ischemic time, median [h:min]	02:52	03:15	0.218	–	–	–
Warm ischemic time, median [h:min]	00:27	00:31	0.67	–	–	–
Change of creatinine during first 24 h post-op	0.39 (0.36)	0.38 (0.1)	0.886	−4.37 (4.02)	−3.92 (3.61)	0.769
Change of creatinine during first 48 h post-op [*n* = 4 vs *n* = 3]	0.58 (0.48)	0.5	0.446	−5.35 (5.07)	−5.78 (6.6)	0.897
Dialysis post-op, *n* [%]	0 (0)	0 (0)	1	0 (0)	0 (0)	1
Urinary NGAL pre-op, median [QR]	9 (13.3)	9 (9.5)	0.906	521.65 (660.1)	421.0 (1725.1)	0.497
Change of NGAL 6 h post-op, median (QR) [µg/L]	–	–	–	460.8 (547.2)	822.4 (1219.95)	0.084
Change of NGAL 12 h post-op [µg/L]	–	–	–	447.8 (606.6)	836.7 (1610.45)	0.112
Change of NGAL 24 h post-op [µg/L]	–	–	–	471.35 (1219.95)	842.4 (1915.63)	0.125
Cystatine-C preop, mean (SD)	–	–	–	5.12 (1.07)	5.67 (1.63)	0.581
Change of Cystatine-C 24 h post-op, mean (SD)	–	–	–	−3.32 (0.85)	−3.93 (1.22)	0.429
Change of Cystatine-C 48 h post-op, mean (SD)	–	–	–	−3.32 (0.85)	−3.97 (1.22)	0.302
AKI during first 7 days postop, *n* [%]	6 (100)	6 (100)	1	0 (0)	1 (16.67)	0.341
Max. creatinine until discharge, median (QR) [mg/dL]	1.57 (0.69)	1.39 (0.76)	0.652	5.725 (4.78)	5.385 (6.33)	0.893
Rejection, *n* [%]	–	–	–	2 (0)	2 (1)	0.549
Duration of hospital stay, median (QR) [d]	9.5 (2.5)	9.5 (4.75)	0.757	22.5 (7)	23.0 (8)	0.167
Change of donor’s creatinine preparation to pre-op (preop - first consultation) [mg/dL]	−0.005 (0.11)	0.025 (0.1)	0.731	–	–	–
Change of donor’s creatinine 12 months after Tx (12 months - preop), median (QR) [mg/dL]	0.39 (0.27)	0.34 (0.27)	0.382	–	–	–

*y* years, *Q* quartile, *QR* interquartile range, *SD* standard deviation.

### Molecular outcome

#### Lipidomic analyses of perirenal fat

Considering the significant weight loss observed in the CR group, we went on to examine metabolic changes in adipose tissue. By untargeted normal-phase chromatography, lipidomic features have to be measured in different modes according to their polarities – i.e. positive or negative. 9913 features could be identified in the positive mode and 2977 features in the negative mode across all samples. Statistical analysis identified 33 features with a *p*-value < 0.01 and a minimal log2foldchange of ≥2 in the positive mode (Fig. 1C). To make sure that all these measurements are reliable the chromatograms of these 33 features were manually reviewed. Afterwards, 21 features were considered plausible. Ten features were downregulated and 11 were upregulated. Nine out of 10 of the downregulated positive features were triacylglyerides (TAG) whereas 7 out of 11 upregulated positive features were diacylglyerides (DAG) (Supplementary Data S1). The Mummichog pathway analysis (Supplementary Data S2) suggests a probable enrichment of the zymosterol biosynthesis (Fig. 1D). Features detected in the negative mode (Supplementary Data S3) as well as additional hydrophilic interaction liquid chromatography (HILIC, Supplementary Data S4) did not reach statistical significance after manual review of the chromatograms.

#### Proteomic analysis of blood serum samples

**Fig. 2:**
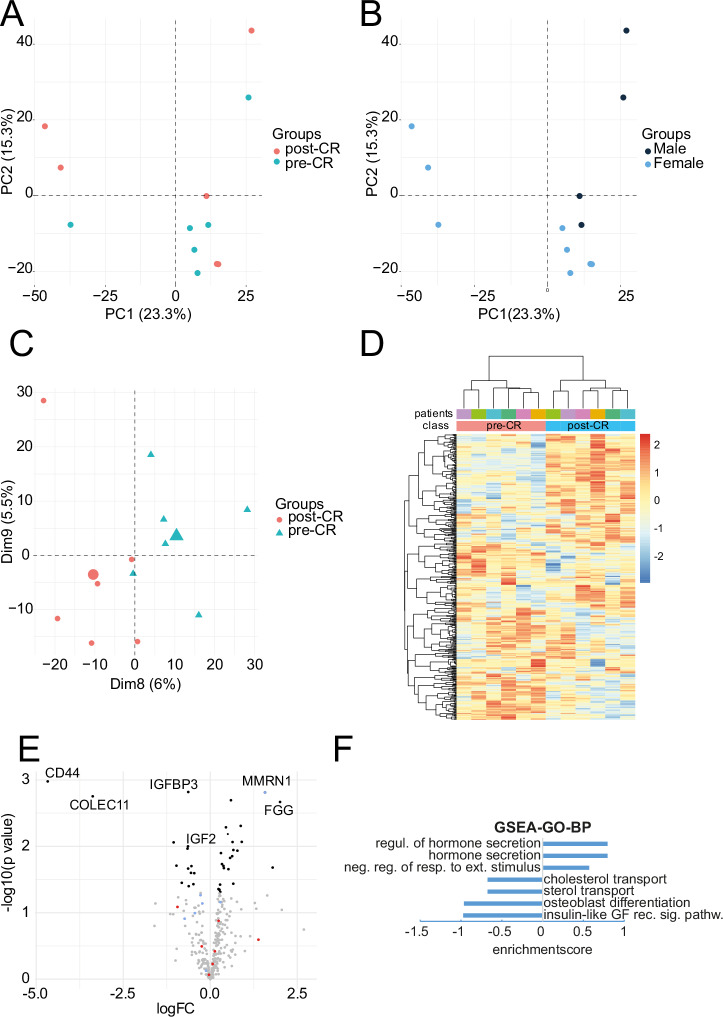
Proteomic analyses of longitudinally acquired blood serum samples of 6 individuals. **A** Principal component analysis showing PC1 and PC2 regarding groups pre- and post-CR. (*n* = 6 per group) **B** Principal component analysis of the same samples depicted in (**A**). showing PC1 and PC2 regarding sex differences of the individuals (*n* = 6 per group). **C** Principal component analysis showing PC8 and PC9 of the same samples as in (**A** and **B**) regarding groups pre- and post-CR (*n* = 6 per group). **D** Heatmap and z-score based hierarchical clustering of the blood serum samples regarding matched pairs of longitudinally acquired blood samples (*n* = 6 per group). **E** Volcano plot of the serum proteins in comparison post-/pre-CR. gray: *p*-value > 0.05; black: *p*-value < 0.05; red dots: proteins known from a former murine transcriptional analysis (Johnsen M et al. JASN 2020, 31(4):p 716-730); blue dots: proteins known from a former murine proteomic analysis (Koehler FC et al. Translational Research 2022; 244:32 46) **F** Geneset enrichment analysis of GO-BP-pathways (*p*-value < 0.0015, *q* ≥ 0.24) CR calorie restriction.

To characterize the molecular effect of CR on the level of serum protein content, we performed label-free proteomic analysis of the longitudinally acquired blood-serum samples collected at screening and in the morning of the day of kidney donation. We identified a total of 907 proteins and 309 proteins were measured across all samples. In a principal component analysis (PCA), dimensions 1 and 2 did not clearly separate samples by dietary intervention, i.e. pre- and post-CR (Fig. 2A), but by sex (Fig. 2B). Samples pre- and post-CR were separated by components 8 and 9 indicating the large impact of other factors on serum proteome composition in heterogeneous human cohorts (Fig. 2C). Z-score-based hierarchical clustering, that takes a longitudinally (i.e. paired) sample-acquisition into account, revealed a clear separation of samples pre- vs. post-CR (Fig. 2D). As no protein remained significant after correction for multiple testing (adjusted *p*-value < 0.05) in the comparison of post- versus pre-CR protein abundance, we continued the analysis based on unadjusted *p*-values to allow for comparison with previous datasets from rodent models and considering the exploratory nature of this study. Using a *p*-value cutoff of <0.05 we identified 24 proteins with increased and 14 with decreased abundance (Supplementary Data S5). Most strongly changed were CD44 (log2FC: −4.67, *p*-value: 0.001), COLEC11 (log2FC: −3.8, *p*-value: 0.0017), both involved in inflammatory processes and MMRN1 (log2FC: 1.57, *p*-value 0.0015) and FGG (log2FC: 2.01; *p*-value: 0.002) both involved in coagulation functions. Furthermore, IGFBP3 and IGF2 as candidates involved in insulin-like growth factor signaling were less abundant (log2FC < −0.6, *p*-value: 0.01). Comparing this dataset with former datasets of our group we identified 7 transcripts (Supplementary Data S5)^17^ and 7 proteins (Supplementary Data S5)^11^ that had been detected in mice as differentially regulated in kidneys of calorically restricted mice to controls (Fig. 2E). In the present dataset Multimerin-1 (MMRN1) and Plexin Domain Containing 2 (PLDXDC2) were the most strongly regulated candidates known from our previous mouse studies on CR^11,17^. MMRN1 showed a trend to an increased abundance (log2FC 1,57; *p*-value: 0.001), but the abundance of PLDXDC2 was only slightly changed (log2FC: −0.94; *p*-value: 0.08) respectively (Supplementary Data S5). Using log-fold changes in protein abundance as input, a gene set enrichment analysis (GSEA) of gene ontology biological process (GO-BP) terms (*p* < 0.0015) showed that after CR proteins related to the pathways “regulation of hormone secretion”, “hormone regulation” and “negative response to external stimulus” were increased and proteins related to “Insulin-like growth factor signaling”, “cholesterol transport”, as well as “sterol transport” and “osteoblast differentiation” were decreased (Fig. 2F). GSEA of GO molecular function (GO-MF, *p* < 0.05) showed an upregulation of “liposaccharide binding”, “transition metal ion binding”, “endopeptidase activity”, “catalytic activity” and a downregulation of “protein-liquid complex binding”, “lipoprotein particle binding”, “high-density lipoprotein binding”, “apolipoprotein receptor binding” and “actin filament binding” (Supplementary Fig. S2A). Additional GSEA of GO-terms of cellular components (GO-CC, *p*-value < 0.05) showed an upregulation of “transferase complex”, “blood microparticle”, “plasma membrane” and a downregulation of “nucleus”, “plasma membrane bounded cell projection”, “apical part of cell”, “actin-based cell projection”, “basal plasma membrane”, “apical plasma membrane”, “leading edge membrane” (Supplementary Fig. S2B).

#### Proteomic analysis of kidney biopsies

**Fig. 3:**
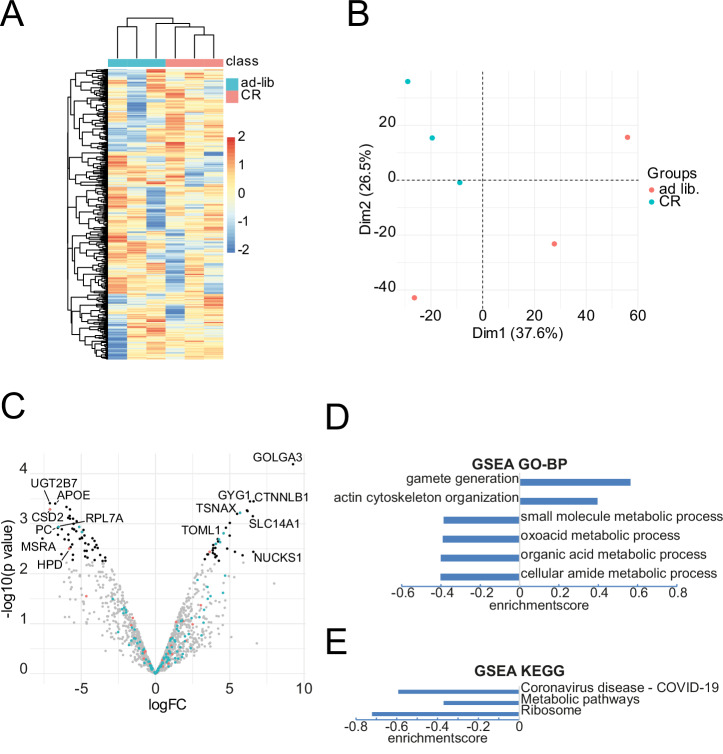
Proteomic analyses of kidney biopsies. **A** Heatmap and z-score based hierarchical clustering (*n* = 3 per group). **B** Principal component analyses (*n* = 3 per group). **C** Volcano plot comparing kidney biopsy samples CR vs. ad libitum. gray: *p*adj >0.1; black: *p*adj <0.1; red dots: proteins known from a former murine transcriptional analysis (Johnsen M et al. JASN 2020, 31(4):p 716-730); green dots: proteins known from a former murine proteomic analysis (Koehler FC et al. Translational Research 2022; 244:32 46). **D** Geneset enrichment analysis of GO-BP-pathways (*p*-value < 0.0004; *q*-value < 0.05). **E** Geneset enrichment analysis of KEGG-pathways (*p*-value < 0.0003; *q*-value < 0.015). ad lib. ad libitum, CR calorie restriction.

To further evaluate the CR-mediated effects on kidney tissue label-free proteomic analysis of kidney null-biopsies directly after nephrectomy was performed. 1511 proteins were identified across all samples. Group separation in kidney proteomics by intervention was much clearer than in the serum proteome datasets. Both, Z-score based hierarchical clustering and PCA showed a clear separation into treatment groups (Fig. 3A/B). A differential analysis of protein abundance revealed 80 significantly changed proteins (*p*adj <0.1, Supplementary Data S6). The most strongly less abundant proteins were methionine sulfoxide reductase (MSRA) (log2FC −7.6), UDP-glycosyltransferase (UGT2B7) (log2FC: −7.1), CDGSH iron sulfur domain 2 (CISD2) (log2FC: −7.1), apolipoprotein E (APOE) (log2FC: −6.7) and pyruvate carboxylase (PC) (log2-6.5) all involved in metabolic processes. The most strongly upregulated proteins with a *p*-value < 0.05 were golgin A3 (GOLGA3) (log2FC: 9.2), β-Catenin (CTNNLB1) (log2FC: 6.6), nuclear casein kinase and cyclin-dependent kinase substrate 1(NUCKS1) (log2FC: 6.58), solute carrier family 14 member 1 (SLC14A1) (log2FC: 6.5) and glycogenin-1 (GYG1) (log2FC: 6.3).

Interestingly, of these proteins, 108 transcripts^17^ and 36 proteins^11^ had been reported in former datasets of our group (Fig. 3C and Supplementary Data S6). The proteins with the most decreased abundance, already known from former transcriptomics of murine kidneys^17^, were ribosomal protein L7a (RPL7A) (log2FC: −6.5), coiled-coil domain-containing protein 93 (CCDC93) (log2FC: −5.1), ribosomal protein S6 (RPS6) (log2FC: −4.9), rippling muscle disease 1 protein (RMD1) (log2FC: −2.88) and syntaxin-8 *(*STX8) (log2FC: −2.1). The corresponding most strongly upregulated proteins were translin-associated protein X (TSNAX) (log2FC: 5696), glycolipid ransfer protein (GLTP) (log2FC: 2827), glutathione S-transferase Mu 1 (GSTM1) (log2FC: 4.69), TOM1-like protein 1 *(*TOM1L1) (log2FC: 4.58) and laminin-gamma-1 (LAMC1) (log2FC: 4.54).

The most strongly changed proteins known from former murine proteomics^11^ (*p*-value < 0.05) were superoxide-dismutase (Cu-Zn) (CSD2) (log2FC: −7.1), 4-hydroxyphenylpyruvate dioxygenase (HPD) (log2FC: −5.8), carbonyl reductase 1 (CBR1) (log2FC: −4.6), fibrinogen beta chain (FGB) (log2FC: 3.05), myelin expression factor-2 (MYEF2) (log2FC: 3.6) and syntaxin-8 (STX8) (log2FC: −2.1). STX8 was also known from the murine transcriptomics^17^. Gene set enrichment analyses of GO-BP terms (*p*-value < 0.0001; *q*-value < 0.05) showed that after CR ”gamete generation” and “actin cytoskeleton organization” were enriched and terms related to cellular metabolic processes as “small molecule metabolic process”, “oxoacid metabolic process”, “organic acid metabolic process” and “cellular amid metabolic process” were decreased (Fig. 3D). Further GSEA of KEGG-pathways showed that “Coronavirus disease – COVID-19”, “metabolic pathways” and “Ribosome” were decreased (Fig. 3E, (*p*-value < 0.0015, *q* ≥ 0.24)). GO-enrichment analysis of term concerning molecular function revealed “carboxylic acid binding”, “structural constituent of ribosome”, “catalytic activity, acting on RNA”, “RNA binding” and “nucleic acid binding” as mostly enriched (all *p*-value < 0.05 and at least 5 proteins annotated, *q*-value < 0.8) (Supplementary Fig. S3).

#### Cytokine/chemokine-analysis

As nutrition is known to modulate systemic inflammation and we detected the downregulation of the KEGG term “Coronavirus disease – COVID-19” in kidney biopsies, representing systemic inflammation, we quantified concentrations of 50 proteins including cytokines, chemokines, growth factors, and endothelial factors. We analyzed kidney donors’ blood serum samples pre- and post-CR as well as renal arteries and ureter specimen comparing CR and *ad libitum* directly after nephrectomy.

**Fig. 4:**
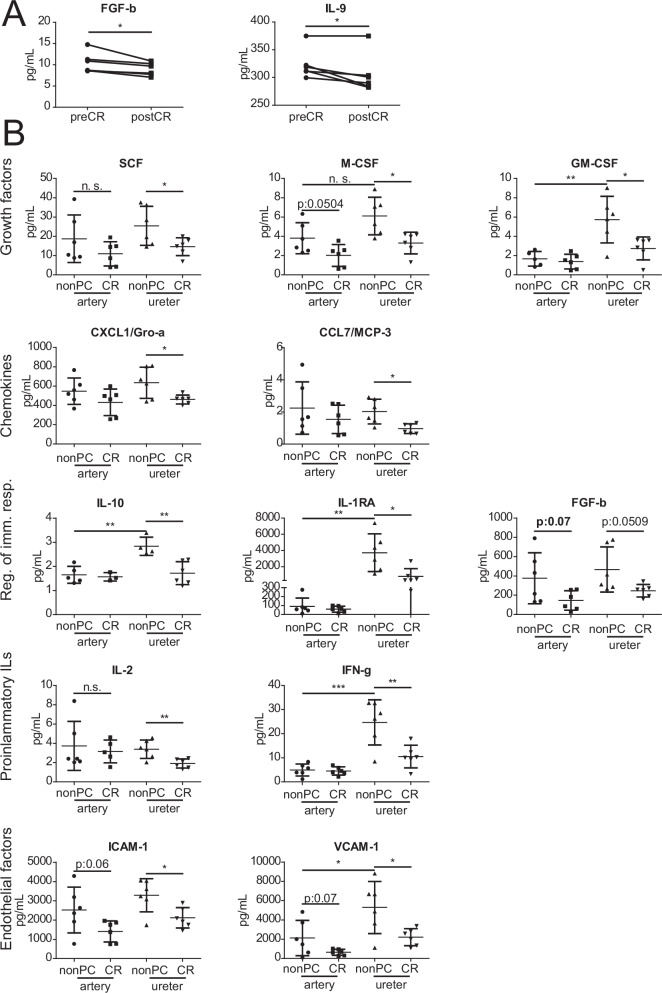
Cytokine analyses of blood serum, renal artery and ureter specimen. **A** Blood serum concentrations of significantly altered cytokines measured pre- and post-CR (*n* = 6 per group, *p* < 0.05). **B** Tissue concentrations in renal artery and ureter specimen of significantly altered cytokines measured in non-preconditioned vs. calorically restricted patients (*n* = 6 per group, *p* < 0.05). nonPC non-preconditioned, CR caloric restriction, n.s. *p*-value > 0.05; *p-value < 0.05; **p-value < 0.01; ***p-value < 0.001.

All 50 proteins (cytokines, chemokines, growth factors) were reliably quantified in all samples. As expected, in healthy kidney donors prior to kidney transplantation, analyses of blood serum samples did not show high levels of inflammation or endothelial activation, but interestingly, after CR we detected significant decreases of fibroblast growth actor beta (FGF-b) and interleukin-9 (IL-9) (Fig. 4A). The concentrations of the other 48 proteins were not significantly changed after CR (Supplementary Data S7).

Comparing protein concentrations of artery and ureter specimen out of the control group, they differed significantly in 11 proteins. In artery specimens, tumor-necrosis factor beta (TNF-β/lymphotoxin-α) and interferon-gamma inducible protein 10 (CXCL10/IP-10) showed significantly higher concentrations than in ureter samples. In ureter samples interleukin-18, interleukin-13, interleukin-10, interleukin-15, interferon gamma (IFN-γ), macrophage colony-stimulating factor (M-CSF), granulocyte-macrophage colony-stimulating factor (GM-CSF), interleukin-1-Receptor antagonist (IL1RA) and vascular adhesion molecule 1 (VCAM-1) showed significantly higher concentrations than in artery specimen. After CR we detected significantly lower concentrations in comparison to controls of growth factors (SCF, M-CSF and GM-CSF), chemokines (CXCL1/GRO-a, CCL7/MCP-3), regulators of the immune response (IL-10, IL1RA), proinflammatory interleukins (IL-2, IFN-γ) and endothelial adhesion molecules (VCAM-1, ICAM-1) in ureter specimen. Although it did not reach statistical significance, there was a noticeable trend of lower concentrations of the same proteins in artery specimen after CR (Fig. 4B). Taken together, inflammatory cytokines, chemokines and growth factors differed between artery and ureter vessels and were partially reduced upon CR, indicating that the local environment in renal vessels can be modulated by nutrition.

## Discussion

Renal ischemia-reperfusion injury can be reliably reduced by short-term CR in animal models as we and others have unequivocally demonstrated^8–11,17^. Nevertheless, translation into the clinical setting remains scarce. Only a few trials have been conducted in the setting of kidney transplantation as a prime example of renal IRI. While van Ginhoven et al. provided first evidence in 2011 that a short-term CR over 3 days prior to surgery is safe and feasible in the setting of living kidney donation^15^, Jongbloed et al. showed 2020 that a combined protein and calorie restriction over five days prior to surgery may improve outcomes after living kidney donation^14^. To our knowledge, the here presented trial is the first with a primary focus on the molecular effects after 50% CR of the calculated DEE over seven days of CR prior to surgery.

The dietary intervention over one week was well tolerated. All patients of the intervention group stayed on the CR-diet as defined by the protocol, and only some of the patients reported harder stools at the second visit, but without signs of severe obstipation. As reported in previous trials, also patients of the control group showed a reduced calorie intake (75%) in comparison to the calculated DEE^18^ probably influenced by the information about positive effects influenced due to preoperative weight reduction. Medical intervention due to trial-related adverse events was not needed in any situation. Patients of the intervention group showed a significant weight loss, but there was no significant effect detectable neither on the development of AKI during the first 7 days post-surgery, the change of urinary NGAL or serum cystatin-C nor the length of hospital stay. These results on clinical endpoints were expected as the clinical outcome after living kidney transplantation is usually very good due to the smaller impact of IRI and DGF compared to kidney transplantation of deceased donors^19,20^. Furthermore, strong effects on clinical endpoints were not expected as this trial was designed to address molecular changes as its primary endpoint and was not powered to stratify e.g. between pre-emptive or recipients already receiving chronic dialysis therapy with consecutively reduced urine production influencing NGAL-values in the urine. Additionally, the unintended reduced caloric intake of the control group due to reduced between-group differences can be regarded as an effect size diminishing confounder resulting in undetectable effects on the clinical outcome.

On the contrary, there was a significant impact detectable on the molecular level. The lipidomic analysis of perirenal fat showed a decrease of triacylglycerides and an increase of diacylglycerides as a result of an activated lipolysis. From the clinical point of view this is, in addition to the weight change, explicit evidence for adherence to the dietary intervention. This finding also goes in line with previous studies that clearly showed a CR-mediated activation of lipolysis and activation of lipid metabolic processes. The detected overrepresentation of “zymosterol biosynthesis” seems quite reasonable as zymosterol is an intermediate product in cholesterol biosynthesis, a process previously implicated in response to CR^21^.

In line with results of Jongbloed et al.^14^, the proteomic serum blood analysis at first demonstrated effects mainly mediated by differences in sex. A robust sex-specific subgroup analysis was not possible due to the limited sample size. However, after the integration of the influencing factor sex into the linear data analysis model, the analysis revealed a decrease of insulin-like growth factor receptor signaling and a change of sterol transport. Both are very plausible in the context of CR^8,9,17,22^. As major nutrient-sensing pathways increased insulin sensitivity and reduced insulin-like growth factor (IGF) signaling have been linked before to reduced cellular senescence, increased mitochondrial function as well as increased stem cell function resulting in improved organic stress resistance in mice and men^23–26^. Furthermore, a reduced cholesterol transport mediated by CR-induced activation of sirtuins has been described before^23,27,28^ and can be linked to the activation of triacylglyceride catabolism described above^28–30^.

As expected, more robust changes were observed on the level of the kidney proteome. CR resulted in an upregulation organization of the cytoskeleton, reduced metabolic processes and, in contrast to the blood serum analyses but highly consistent to former studies, to a reduction of complement factors (e.g. CFB and CFD, Fig. 3E & Supplementary Data S6) resulting in a downregulation of inflammatory pathways^8,22,31^. Of note, the strongest downregulation was detected concerning the KEGG-ribosome pathway. This is an important finding directly going in line with a former hallmark study demonstrating short-term CR to effectively reduce nucleolar size in drosophila, mice and humans, strongly connected to a reduction of ribosomal activity and proteostasis directly resulting in longevity^5^. In this trial Tiku et al. demonstrated that several different models of reduced insulin/IGF-signaling as well as inhibition of the mammalian target or rapamycin (mTOR), another well-known nutrient-sensing pathway^23,28^ and regulator of ribosomal biogenesis^32^, lead to an increased lifespan, and that the known CR-mediated key pathways are conserved in worms, mice, flies and humans^5^.

Interestingly, in both proteomic datasets, from blood serum samples and kidney biopsies, proteins such as glutathione S-transferase-1 (GSTM1), known from murine studies^11,17^ to be regulated by CR, could be detected. GSTM1 was increased in kidney biopsies of CR-treated patients as well as in CR-treated murine kidneys^17^ and is involved in the defense of oxidative stress in mice^33,34^, monkeys^35^ and humans^36^. The defense of reactive oxygen species (ROS) has been shown to be one pillar of cellular defense against tubular damage in AKI and can be mediated by CR^17,37^. The strongest downregulated protein known from our former proteomic dataset^11^ is iron-sulfur domain-containing protein 2 (CISD2), and it was shown to be a regulator of autophagy. Autophagy is a mechanism to provide functioning cellular homeostasis and was found to be commonly mediated by a set of different nephroprotective dietary interventions, including CR, in the context of diet-induced kidney protection in rodents^11,38^. Although not all proteins known from former studies were significantly regulated in all studies, considering the higher heterogeneity in human cohorts, it indicates that similar molecular patterns are influenced by CR also in humans.

As expected, cytokine analyses of donors’ blood serum samples did not show high concentrations of pro-inflammatory cytokines or proteins regulating the immune response. Even more interesting was that the paired blood sample analyses revealed a significant decrease of FGF-b and IL-9 after CR. FGF-b is directly involved at the beginning of the PI3K-pathway, and IL-9 is found at the start of the JAK-STAT pathway. Both pathways have been described to be downregulated in the context of CR^23,39,40^ and both pathways are known to regulate the immune response^41,42^. Due to the anatomical location of ureters and thereby higher pathogen exposure, it is comprehensible that ureters showed a more differentiated cytokine profile with higher levels of regulators of the immune response, e.g. IL1RA, than renal arteries of the control group.

Of note, levels of proinflammatory cytokines (e.g. IL-2, IFN-g) and regulators of the immune response (e.g. IL-10, IL-1RA), growth factors (e.g. SCF, M-CSF), chemokines (e.g. CXCL1/Gro-a, CCL7/MCP-3) as well as endothelial adhesion molecules (e.g. ICAM-1, VCAM-1) were all significantly lower in ureters of the CR group pointing towards an CR-induced holistic immunomodulatory effect by reduction of circulating immune cells as well as inflammatory mediators^43^. Intriguingly, the analyses of renal arteries in the CR-group showed the same trend of exactly the same proteins in comparison to controls. Altogether, these results reflect another brick pointing towards a systemic CR-mediated response to a second stimulus such as IRI in humans and show that targeted modulation of cytokine levels might play a role in future interventional studies.

This study uses a multiomics approach in a cohort with limited sample size. Consequently, the results should be considered exploratory in nature and as this trial focused on CR prior to living kidney donations, the transferability on kidney transplantations of elderly, multimorbid patients or transplantations of deceased donors remains scarce. Nevertheless, this trial clearly demonstrates that CR has holistic impact which is not restricted to the kidneys. For the future, it is interesting to test in a larger study population, if CR in kidney recipients also shows similar molecular effects or even has preventive impact on clinical endpoints – e.g. reduced delayed graft function. Additionally, CR prior to deceased kidney donation seems hardly achievable, but could be translated to druggable approaches.

In summary, this proof-of-principle study shows that short-term CR prior to living-kidney kidney transplantation is safe and feasible. Most importantly, the results support the concept that CR in humans mediates protective mechanisms such as catabolism of triacylglycerides, reduced insulin signaling, immune tolerance and ribosomal stabilization consistently with the findings from experiments in rodents. Consequently, this study encourages future larger trials addressing the sex-mediated influence on the one hand and detecting effects on clinical endpoints such as transplant function on the other hand. Ideally, future trials should also address the scenario of kidney transplantations of deceased donors. Considering the broad implications of CR-induced stress resistance in different species and organs, these findings come with important implications for aging-associated disease beyond kidney injury. Furthermore, future animal models pharmacologically influencing the pathways and cytokines overlapping between species will add important information on the way to clinical translation.

## Methods

### Study population

This pilot trial was initiated and conducted as an open-label single-center study at the University Hospital Cologne. Approval was obtained from the institutional review board of the University Hospital Cologne (approval Number: 15-031) and the trial was registered on clinicaltrials.gov (NCT02745444, Registration date: 15 April 2016) as well as the German Register for Clinical Trials (DRKS00007704, Registration date: 14 October 2015).

Patients (aged ≥18 years, BMI ≥ 18.5 kg/m²) scheduled for living kidney transplantation were enrolled after written informed consent. There were no exclusion criteria except the inability to give informed consent. The study was conducted in accordance with the Declaration of Helsinki and the good clinical practice guidelines by the International Conference on Harmonization. The trial protocol is provided in the Supplemental Material – Protocol S1.

### Dietary intervention and sample collection

Patients in the CR group were provided with a formula diet (Fresubin energy fiber drink, Fresenius Kabi Deutschland GmbH, Bad Homburg, Germany) that contained all necessary macro- and micronutrients. The amount was limited to provide only 50% of the daily energy expenditure (DEE), as calculated using the Mifflin–St. Jeor equation and individually assessed activity factors. Participants were instructed not to consume extra food or calorie-containing beverages like fruit juices, alcohol or soft drinks. The CR started on preoperative day −7 and was maintained until the patients were instructed to stop eating and drinking due to the planned anesthesia in the evening of day −1. On the day of surgery, patients were maintained in a fasting state until surgery. In the control group, patients were allowed to ingest food as they were used to do. All participants in both groups were provided with diaries and reported their food consumption on a daily basis.

Reassessment of all participants was performed on pre-operative day −1 (visit 2). Blood serum and urine samples were collected in the morning of the day of surgery before start of the anesthesia as well as within the postoperative times defined in the trial protocol. Perirenal fat was acquired directly after nephrectomy. Kidney biopsy samples for proteomic analysis as well as renal artery and ureter specimens were obtained directly after saline/heparin perfusion before replantation or reperfusion. All laboratory parameters after surgery as well as type of surgery or ischemia-times were extracted from the medical records. Baseline demographic and clinical data including comorbidities and medication were assessed at the screening visits. Anthropometric data (e.g. body weight, body composition) were recorded at the screening visits and at hospital admittance. All patients were followed up until their hospital discharge.

### Surgical procedure

Surgical procedures were not influenced by the trial protocol and were performed according to the local standard. Briefly, nephrectomy was performed using the hand-assisted retroperitoneoscopic (HARP) technique as previously described^16^.

### Statistical analysis

For clinical statistical analyses IBM SPSS Statistics version 29 (IBM Deutschland GmbH, Böblingen, Germany) was used. For descriptive statistics “explorative data analysis” was used to calculate means and interquartile ranges. For differences between two groups two-sided Student’s *t* tests were calculated and a *p*-value < 0.05 was considered significant. For analyses of more than two groups, one-way analyses of variance (ANOVA) with Tukey posthoc test were performed. For lipidomic and proteomic analyses, if not otherwise mentioned, *p*-values < 0.1 were considered significant.

### Lipidomics

Untargeted lipidomics was performed given a modified method previously described by Breitkopf and colleagues^44^. Snap frozen fat tissue was weighed in and lipids were extracted using the Folch method, using 2:1 chloroform:methanol. Samples were analyzed on a Quadrupole-time-of-flight instrument (Impact II, Bruker, Bremen, Germany). The chromatography was performed by a coupled UHLPC device (Bruker Elute, Bruker, Billerica, MA, USA). Data was acquired over a m/z range 50–1200 Da in positive ion mode, and negative ion mode, respectively. Mass spectrometer calibration was performed using Sodium formate (post-run mass calibration). The electrospray source conditions were: end plate offset was 500 V, dry gas temperature, 200 °C, drying gas 6 L/min, nebulizer was 1.6 bar. Capillary voltage was set to 3500 V. A dual fractionation strategy was used to increase metabolome coverage and minimize ion suppression. RP chromatography was done on an ACQUITY BEH C18 column (1.0 × 100 mm, 1.7 μm particle size, Water Corporation, Milford, MA) Buffer A pH = 3.5: 39.9% (v/v) water, 60% (v/v) acetonitrile, 10 mM ammonium formate, 0.1% formic acid. Buffer B: 89.9% (v/v) isopropanol, 10.0% (v/v) acetonitrile, 10 mM ammonium formate, 0.1% formic acid. Differential lipid species and peaks were detected by XCMS online using the following parameters:positive mode 10 ppm maximal tolerated m/z deviation, minimum peak width = 5, mximum peak with = 20, Signal/noice threshold = 6, mzdiff = 0.01, integration method = 1, prefilter peaks = 3, prefilter intensity = 100, noise filter = 100, obiwarp retention time alignment. Statistically selected peaks were annotated using Lipidmaps and Metlin, followed by auto-MS and fragmentation at 10, 20 and 40 eV using an inclusion list. Annotations were manually validated using known fragmentation patterns for lipids, as well as m/z v values (lipidmaps^45^, metlin^46^).

### Sample preparation for blood serum proteomics

Samples were prepared according to a modified SP3 protocol^47^ on a Chronect Robotic RSI (Axel Semrau, Sprockhövel, Germany) and an Integra Assist plus (Integra Biosciences, Zizers, Switzerland), omitting peptide purification on the second day. Instead, beads were removed after acidification and samples were either purified using mixed-mode StageTips or loaded directly onto Evotips following the manufacturer’s recommended protocol.

Samples were analyzed using either an EASY 1200 nLC coupled to a Q Exactive HFx (both Thermo Fisher Scientific, Bremen, Germany) or an Evosep One (Evosep, Odense, Denmark) coupled to an Orbitrap Exploris 480 with FAIMS pro (both Thermo Fisher Scientific, Bremen, Germany).

### Sample preparation for kidney proteomics

Samples were analyzed on an Orbitrap Exploris 480 equipped with FAIMS pro (both Fisher Scientific, Schwerte, Germany) coupled to an Evosep One nLC (Evosep, Odense, Denmark). Samples were chromatographically separated using the Whisper 20 SPD method using a Pepsep column with the recommended specifications and a 10 µm glass emitter. FAIMS was run at a constant CV of −50 with the inner and outer electrode heated to 99.5 °C and 85 °C, respectively, and 3.7 slm gas flow. The Exploris was run in data-independent acquisition mode in the range of 400–1000 m/z using staggered isolation windows with a width of 20 m/z and shifted by 10 m/z for every second cycle. MS1 scans were acquired before each cycle with a resolution of 45k, AGC target of 100%, and a scan range of 380 to 1020 m/z. MS2 scans were acquired with 45k resolution and an AGC-target of 1000%.

### Data processing protocol for proteomic analyses of blood serum samples

Samples were converted into htrms files and then processed in Spectronaut 18.7 (Biognosys, Schlieren, Switzerland) in directDIA mode with standard settings using the Uniprot canonical Human reference proteome (UP5640, downloaded 4/1/2024). Afterwards, identified and quantified protein groups were exported and analysis was performed on the log2 intensity values after filtering the dataset to exclude keratins. Subsequent heatmap visualization and hierarchical clustering were carried out on Z-score-transformed data using the R package pheatmap (version 1.0.12)^48^. PCA was performed using the nsprcomp function from the R package nsprcomp (version 0.5.1-2)^49^.

Differential expression analysis was restricted to proteins detected in all samples (309 proteins out of 886 proteins) and performed using the limma package (version 3.56.2)^50^.

For gene set enrichment analysis (GSEA), the functions gseGO and gseKEGG from the R package clusterProfiler (version 4.11.1)^51^ were used to identify enriched GO terms and KEGG pathways, respectively.

### Data processing protocol for proteomic analyses of kidney biopsies samples

Raw files were demultiplexed using MSConvert from the ProteoWizard package^52^ resulting in nominal 10 m/z windows. Samples were processed in DIA-NN 1.8.1^53^ using the MBR function to directly generate a spectral library based on analyzed samples and the canonical Human Uniprot reference proteome (UP5640, downloaded 13/01/2022). DIA-NN was run with the additional command line prompts “—report-lib-info” and “—relaxed-prot-inf”. Further output settings were: filtered at 0.01 FDR, N-terminal methionine excision enabled, maximum number of missed cleavages set to 1, min peptide length set to 7, max peptide length set to 30, min precursor m/z set to 400, max precursor m/z set to 1000, cysteine carbamidomethylation enabled as a fixed modification. Afterwards, DIA-NN output was further filtered on library q-value and global *q*-value ≤0.01 and at least two unique peptides per protein using R (4.1.3). Finally, LFQ values were calculated using the DIA-NN R-package. Afterwards, analysis of results was performed in Perseus 1.6.15^54^. The imputed data were exported from Perseus and the log2 LFQ values were used for statistical analysis.

The data were median-centered by subtracting the median value from each observation.

Hierarchical clustering and heatmap visualization were performed on Z-score-transformed data using the pheatmap package in R (version 1.0.12)^48^. Principal component analysis (PCA) was conducted with the nsprcomp function from the R package nsprcomp (version 0.5.1-2)^49^.

Differential expression analysis was carried out using the limma package in R (version 3.56.2)^50^. Functional enrichment analysis for Gene Ontology (GO) terms was performed using the enrichGO function from the R package clusterProfiler (version 4.11.1)^51^.

### Cytokine, chemokine and growth factor quantification in plasma and tissue samples

Cytokine, chemokine and growth factor concentrations in donor serum were quantified by the Luminex-based multiplex technique according to the manufacturer’s instructions (Bio-Rad, USA). The Human 48-Plex (#12007283M), plus soluble ICAM-1 (#171B6009M) and soluble VCAM-1 ((#171B6022M, all from Bio-Rad, Hercules, USA) were combined in one 50-Plex assay and serum samples were diluted 1 + 1 with sample diluent. Protein lysates were generated from snap-frozen tissue samples of donor renal artery and ureter vessels (Bio-Rad Cell Lysis Kit #171304011 Bio-Rad, Hercules, USA; modified by protein lysate resuspension in Bio-Plex sample diluent). All protein lysates we adjusted to 50 µg/100 µl and 25 µg/50 µl total protein were used for the 50-Plex assay. All 50 standard curves and respective sample concentrations were calculated with the Bio-Plex Manager 6.2 Manager program (5-parameter logistic regression plot) and the lower detection limit of all 50 proteins was 1–2 pg/ml.

## Supplementary information

Supplementary Information

dataS1_Lipidomics_pos_p0.05_MaxInt15K_2025_07_09

dataS2_mcg_pathwayanalysis_mummichog

dataS3_lipidomics_neg_results

dataS4_HILIC_results

dataS5_serumproteomics_post_vs_pre

dataS6_kidney_biopsie_proteomics_CR_vs_control

dataS7_cytokines

## Data Availability

The datasets generated and/or analyzed during the current study are not publicly available due to ethical and privacy issues, but are available from the corresponding author on reasonable request.
