# Supplementary material for: An in-depth analysis of the molecular changes induced by short-term calorie restriction before living kidney donation
Source: NPJ Aging. 2026 May 28;12(1):70. doi: 10.1038/s41514-026-00401-w (PMC13219448; doi:10.1038/s41514-026-00401-w)
Supplement: Supplementary file 1 — Supplementary Information [file 41514_2026_401_MOESM1_ESM.pdf]

Supplements

ITEM **Page**

ITEM	Page
Protocol S1: Trial protocol	2
Figure S1: Timeline and baseline characteristics.	23
Figure S2: Geneset enrichment analysis (GSEA) of blood serum proteomics.	24
Figure S3: GO-enrichment of GO-MF terms.	25

For supplemental data files please take a look at the separate excel files:

Data S1: Results of lipidomic analysis in the positive detection mode.

Data S2: Mummichog pathway analysis of the detected features in the positive mode.

Data S3: Results of lipidomic analysis in the negative detection mode.

Data S4: Results of hydrophilic interaction liquid chromatography (HILIC).

Data S5: Results of the serum proteomic analysis.

Data S6: Results of the kidney proteomic analysis.

Data S7: Results of the cytokine analysis.

Trial Protocol

Prevention of acute kidney failure

Molecular mechanisms of dietary preconditioning in humans

Application for submission to the Ethics Committee of the University Hospital of Cologne

Version 1 from 29.01.2015

Contact information:

PD Dr. med. Volker Burst

Clinic II for Internal Medicine

Hospital of the University of Cologne

Kerpener Str. 62

50937 Cologne

Phone: 0221 / 478 - 86285

Fax: 0221 / 478 - 5959

E-mail: volker.burst@uk-koeln.de

Synopsis

Responsible manager and principal investigator	PD Dr. med. Volker Burst<br>Clinic II for Internal Medicine<br>University of Cologne<br>Kerpenerstr.62<br>50937
Title of the clinical trial:	Prevention of Acute Renal Failure:<br>Molecular mechanisms of dietary preconditioning in humans
Indication	Prevention of acute kidney failure
Phase:	Other study<br>non-AMG/non-MPG study
Type of examination: Study design / methodology:	Monocentric clinical trial<br>Monocentric, non-blinded, non-randomized intervention study
Number of patients:	At least 5, max. 10 per treatment group (10 - 20 in total)
Primary study objective	Identification of relevant differences in transcriptome, lipidome, metabolome, epigenome, proteome and phosphoproteome in kidney tissue between subjects after one week of calorie restriction vs. control subjects. Studies in living kidney donation.
Target values:	Primary target variable:<br>• Transcriptome<br>• Lipidome<br>• Metabolome<br>• Epigenome<br>• Proteome<br>• Phosphoproteome<br>Secondary targets:<br>• Change in NGAL (µg/l) in urine (preoperative in donor and recipient; 6 h, 12 h and 24 h postoperative in recipient only)<br>• Cystatin C:<br>preoperative: in the recipient<br>postoperative: daily until the seventh postoperative day exclusively in the recipient<br>• Occurrence of an AKI according to KDIGO I, II, III in the recipient<br>• Maximum postoperative serum creatinine value of the recipient during the inpatient stay

Diagnosis and main inclusion criteria:	Diagnosis: Planned living kidney transplantation for end-stage renal failure<br><br>Main inclusion criteria:<br>1. Person capable of giving consent≥ 18 years<br>2. Planned living kidney donation<br>3. Written consent in the case of legal capacity<br>4. BMI ≥ 18.5 kg/m²<br>Main exclusion criteria:<br>none
Name of the measure	Calorie-reduced formula diet - 50% of energy expenditure individually calculated according to Mifflin-St. Jeor formula - (Fresubin® energy fiber Drink, Fresenius Kabi Deutschland GmbH, Bad Homburg, Germany)
Comparative therapy:	Nutrition ad libitum according to the donor's habits.
Duration of therapy:	Diet: Day -7 to day 0 preoperative<br>(Day 0 corresponds to the day of surgery)
Schedule:	The study runs until the desired number of patients is reached. Assignment to the study arms is alternating Expected trial period: 15.02.2015 to 15.02.2017
GCP conformity:	This trial will be conducted in accordance with the current version of the protocol, the internationally recognized guideline Good Clinical Practice (ICH-GCP) including the archiving of essential documents.

Contents

Synopsis 3
Contents 5
A. Formalities 7
A.1 Name of the study project 7
A.2.1 Applicant 7
A.2.2 Responsible medical director 7
A.3 Responsible body 7
A.4 Previous applications 7
A.5 Consent of the Director of Clinic II for Internal Medicine 7
A.6 Financing 8
A.7 Study-related additional costs 8
A.8 Industrial sponsor 8
A.9 Number of patients in multicenter studies 8
A.10 Explanation 8
A.11 Application of radioactive substances or ionizing radiation to humans 8
B. Study description 8
B.1 Scientific objective 8
B.1.1 Background and scientific question 8
B.1.2 Aim of the study 10
B.2 Study design 10
B.2.1 Timetable 10
B.2.2 Methodology 11
B.2.2.1 Data acquisition 11
B.2.2.2 Target values 11
B.2.2.3 Laboratory tests 11
B.2.2.4 Tissue sampling 12
B.2.2.5 Calorie restriction process 13
B.2.2.6 Instrumental examinations 14
B.2.3 Course of study 14
B.3 Intended procedure, stress for the patient 15
B.3.1 Visits 15
B.3.2 Issue of food 15
B.3.3 Calorie restriction 15
B.3.4 Blood/urine sample assay 15
B.3.5 Taking tissue samples 16
B.4 Patient selection 16
B.4.1 Selection of test subjects 16
B.4.2 Inclusion criteria 17
B.4.3 Exclusion criteria 17
B.4.4 Case number calculation 17
B.5 Type of examination 17
B.6 Legal provisions 17
B.7 Non-authorized medicinal products 17
B.8 Complications and risks 17
B.9 Risk-benefit assessment 18
B.10 Interim evaluation and termination criteria 18
B.11 Content of the patient information / declaration of consent 18
B.12 Data protection 19

B.13 Insurance cover 19
C. Abstract 19
1. rationale / motivation and clinical background of your project proposal 19
2 Explicit research question and objective of the study 19
3. ethical issues 20

A. Formalities

A.1 Name of the study project

Prevention of acute kidney failure
Molecular mechanisms of dietary preconditioning in

A.2.1 Applicant

PD Dr. med. Volker Burst

Clinic II for Internal Medicine

Hospital of the University of Cologne

Kerpener Str. 62

50937 Cologne

Phone: 0221 / 478 - 86285

Fax: 0221 / 478 - 5959

E-mail: volker.burst@uk-koeln.de

A.2.2 Responsible medical director

PD Dr. med. Volker Burst

Clinic II for Internal Medicine, University Hospital of Cologne

A.3 Responsible body

Clinic II for Internal Medicine, University Hospital of Cologne

(Director Prof. Dr. Thomas Benzing)

Focus on transplant surgery

(Head: Prof. Dr. Dirk Stippel)

A.4 Previous applications

No previous applications have been submitted.

A.5 Consent of the Director of the Clinic II for Internal Medicine and the Head of Transplant Surgery

See attachments

A.6 Financing
The study is financed by third-party funds.

A.7 Study-related additional costs
Not applicable.

A.8 Industrial sponsor
Not applicable

A.9 Number of patients in multicenter studies
Not applicable.

A. Explanation
I hereby declare to the Ethics Committee of the Faculty of Medicine of the University of Cologne that I guarantee the factual, personnel and organizational requirements of the clinical trials.

PD Dr. Volker Burst

A.11 Application of radioactive substances or ionizing radiation to humans
Not applicable.

B. Study description

B.1 Scientific objective

B.1. Background and scientific question
Conditioning describes a mechanism that enables an organism to better cope with ischemic damage through adaptation processes (Bein et al. Anasthesiol Intensivmed Notfallmed Schmerzther 2010) .

One way of achieving increased resistance to ischaemia is short-term calorie restriction. It has been known for some time that a long-term diet with moderate calorie restriction leads to a

prolongation of life in animals. In recent years, it has also been shown in a series of animal experiments that a short-term diet also results in direct biochemical and cellular adaptation processes that lead to significantly increased resistance to ischemic organ damage. Several research groups were initially able to demonstrate this in a rat model of liver transplantation (van Ginhoven et al. Liver Transpl 2009) . If the donor animals were left fasting 3 days before surgery, 89% of the recipient animals survived after liver transplantation, while all recipient animals that received an organ from donor animals in the control group without a prior diet died as a result of ischemia. In other studies, similar observations were made in models of cerebral (Yu et al. J Neurosci Res 1999) and cardiac ischemia (Ahmet et al. Circulation 2005) .

In 2010, Mitchell et al. were able to show that a four-week calorie reduction of 30% in mice leads to a dramatic reduction in renal ischemia-reperfusion injury (Mitchell et al. Aging Cell 2010) . In the control group, an AKI-associated mortality of 60% was observed after 40 minutes of renal artery clamping. In contrast, 100% of the animals in the diet group survived with significantly less pronounced renal dysfunction. A similar result was found when the animals were allowed to fast (with free drinking) for only the last 3 days before surgery instead of following a diet for several weeks.

The exact mechanisms are not yet fully understood, but it is assumed that calorie restriction primarily leads to a reduction in oxidative stress levels and to a strengthening of the body's own antioxidant defense mechanisms.

Comparable studies in humans are still few and far between. In a clinical study by another working group, it was shown that a one-week preoperative calorie restriction is also feasible in humans (van Ginhoven et al. Clin Transplant 2011) . Our research group is currently conducting two further clinical studies in which the effectiveness of a one-week diet on the course of postoperative acute kidney failure and contrast agent damage to the kidney is being investigated (clinicaltrials.gov, NCT01534364, NCT01879839). In parallel, we were able to show in animal experiments that a 30% calorie restriction has a protective effect on induced renal failure (ischemia-reperfusion model). Transcriptional analyses (RNA sequencing) of the animals also showed that changes in fat metabolism and mitochondrial metabolism or the mitochondrial respiratory chain play a major role in this.

This raises the question of the effects and mechanisms of action of preoperative calorie restriction in humans, which are to be investigated in more detail in this clinical study.

B.1. Aim of the study

The aim of this pilot study is to investigate the molecular mechanisms of renal preconditioning through a one-week calorie restriction for the first time in humans. In addition, the protective effect of preoperative calorie restriction in the donor on graft characteristics and function in the recipient after transplantation will be investigated.

The transcriptome, lipidome, metabolome, epigenome, proteome and phosphoproteome will be analyzed using tissue samples from the donor (kidney, blood vessels, adipose tissue) and blood cells as well as blood plasma/serum and compared between the groups with and without calorie restriction.

In particular, the findings obtained from animal experiments on a possible mechanism of action of nephroprotective calorie restriction are to be verified by this study using human tissue and blood cells and correlated with clinical surrogate parameters for kidney function. The data previously obtained in animal experiments could thus be verified and compared using human samples. The long-term goal of our working group is to identify possible pharmacological targets within individual metabolic pathways that could lead to organoprotection.

B.2 Study design

Monocentric, 2-arm intervention study, not randomized.

Non AMG, non MPG

B.2.1 Timetable

Recruitment and inclusion of study participants are planned for the period from February 15, 2015 to February 15, 2017 (see Table 1). The end of the trial is set for May 31, 2017.

A final report should be available by 31.12.2017. See section B.2.3. for the exact study procedure.

Table 1: Timetable of the study

Inclusion of first patient<br>(first patient first visit, FPFV):	15.02.2015
Inclusion of last patient (LPFV):	15.02.2017
End of examination of the last patient (LPLV):	31.05.2017
Integrated final report:	31.12.2017

B.2.2 Methodology

B.2.2.1 Data acquisition

The data for regular military welfare are registered in pseudonymized form. This applies in particular to:

- Demographic data: Age, height, weight, gender
- Medical history and physical examination
- Test results
- Current medication

B.2.2.2 Target values

The primary targets are the transcriptome, lipidome, metabolome, epigenome, proteome and phosphoproteome of the donors.

Secondary outcomes are defined as the change in neutrophil gelatinase-associated lipocalin (NGAL) in urine and cystatin C in serum, the occurrence of acute renal failure according to KDIGO I, II, III in the recipient and the maximum postoperative serum creatinine value in the recipient during hospitalization.

B.2.2.3 Laboratory tests

To investigate the effect of the preconditioning procedure in comparison to the control group, blood samples from all transplant recipients participating in the study are analyzed directly before surgery and on each day after surgery up to the seventh postoperative day, and urine samples from the transplant recipient are documented up to the second postoperative day. In particular, creatinine levels are determined at regular intervals - daily - to document the progression over time as a surrogate parameter for kidney function.

Preoperative laboratory tests are taken as part of routine clinical preparation wherever possible. In addition, a one-off additional blood sample is taken and urine sample assayed from both the donor and the recipient preoperatively on the day of the operation.

Post-operative blood samples are taken, where possible, via the central venous catheter (CVC) inserted during the operation and generally do not require an additional puncture.

Laboratory parameters include, but are not limited to

- Serum sodium
- Serum potassium
- Serum creatinine

- Serum urea
- Serum uric acid
- Blood count
- C-reactive protein
- Lactate dehydrogenase
- Creatine kinase

The following parameters are determined as additional biomarkers for renal damage:

- Neutrophil gelatinase-associated lipocalin (NGAL) in urine:
  on the day of surgery, both for the donor and the recipient preoperatively and exclusively for the recipient approx. 6 h (+/- 1.5 h), approx. 12 h (+/- 1.5 h) and approx. 24 h (+/- 1.5 h) postoperatively
- Cystatin C
  Preoperative: as part of the clinical routine exclusively for the recipient
  Postoperative: daily up to the seventh postoperative day exclusively for the recipient

B.2.2.4 Tissue sampling

Punch biopsies are taken from the donor organs independently of this clinical examination as part of regular medical care and clinical routine by the transplant surgeon as part of the explantation or transplantation process for histopathological assessment of the donor organ (so-called zero biopsy). Only approx. 70 % of the biopsy specimen is used by the pathology department, leaving excess tissue to be used for these scientific analyses.

Furthermore, as part of the explantation procedure, the kidney is removed en bloc with parts of its perirenal fat capsule and this part of the fatty tissue is dissected off extracorporeally. In addition, surgical dissection of the vascular stump is usually necessary for re-anastomosis, which also results in small amounts of tissue (vessel wall) that are usually disposed of in routine clinical practice.

This supernatant tissue - fat capsule parts and resected vessel parts - will also be used for further investigation of molecular mechanisms.

A 20 ml whole blood sample is taken from the transplant donor twice for cell analysis. The first time before the start of the diet as part of the first visit and on the morning of the day of the operation as part of the above-mentioned preoperative puncture to determine laboratory parameters

Additional tissue material that is not obtained as part of regular medical care and clinical routine by the transplant surgeon during the explantation or transplantation procedure is not obtained in this study.

The processing of tissue samples and blood cells includes the analysis of the transcriptome, lipidome, metabolome, epigenome, proteome and phosphoproteome.

B.2.2.5 Calorie restriction process

After the study participants have been informed and have given their consent, they are contacted and called to the study center before the planned operation.

This is followed by the 1st visit with recording of the anthropometric parameters.

Patients are then given the amount of Fresubin® energy fiber drink for days -7 to -1, calculated individually according to 50% of their energy expenditure. The daily energy expenditure is calculated using the Mifflin-St. Jeor formula:

Man: Gm = 9.99 x weight [kg] + 6.25 x height [cm] - 4.92 x age [years] + 5

Woman: Gw = 9.99 x weight [kg] + 6.25 x height [cm] - 4.92 x age [years] - 161

The total daily metabolic rate is the sum of the basal metabolic rate and the power metabolic rate.

by multiplying the basal metabolic rate by the activity factor (AF):

AF 1,2	No or only minimal physical strain (sitting, lying down)
AF 1.375	light physical strain (corresponding to walking for 2 h/die)
AF 1,550	moderate physical exertion (corresponding to walking for 3 h/die)
AF 1.725	high physical strain (corresponding to walking for 4 h/die)

Patients receive a diet corresponding to: 50/100 x G x AF.

The nutritional drinks are given to the study participants and stored at home at room temperature or in the refrigerator. Patients are given a weekly consumption plan in table form. The possible need to consume individual drinking bottles only proportionally in order to achieve the calculated calorie amount is discussed in detail with the patients. For this purpose, the study participants are given a measuring cup during visit 1. Consumption is carried out independently at home. The study participants are asked about their well-being and dietary adherence through regular phone calls. The study participants are encouraged to write down all the food they eat. For standardization purposes, patients receive a diet diary as part of visit 1. Any drinking bottles not consumed are disposed of by the study participants at the end of the study.

The study participants are informed that only the intake of calorie-free drinks, e.g. in the form of unsweetened teas or water, is permitted.

Study participants in the control group eat according to their usual habits. A nutrition (diet) diary is kept.

The start of the dietary measure takes place on the morning of day -7 before the surgery date. Study participants will be reminded of the start of the diet by telephone a few days beforehand.

B.2.2.6 Instrumental examinations

None planned.

B.2.3 Course of study

After detailed information and consent, the study participants are called in for their first visit to the study center before the planned operation date. Study participants are understood to be both donors and recipients. During the first visit, body weight, body composition and waist circumference are recorded for all study participants.

The donors who undergo calorie restriction are given their food as described above and are once again informed in detail about the study-appropriate application. Furthermore, 20 ml of whole blood will be taken as a comparative control before the diet.

Donors in the control group eat according to their usual habits. All donors are given a diet diary to document their daily food intake. The donors and recipients are then admitted as inpatients before the operation as part of the applicable routine. As part of the inpatient admission, a routine pre-operative blood sample is taken, which includes the measurement of the above-mentioned laboratory parameters - e.g. serum creatinine, CRP, leukocyte count, creatine kinase, etc.

The 2nd visit takes place for the donor on the day before the operation. After another assessment of body weight, body composition and waist circumference and questioning of the study participants regarding diet adherence during the previous 6 days and after acquisition of the diet diaries, the study participants undergo the usual preoperative examinations according to the requirements of visceral surgery. The diet is continued on day -1 under inpatient monitoring until the anesthesiologically required food and fluid restriction in the clinic.

According to the above information, in addition to the routine examinations, all study participants undergo a study-related preoperative urine collection to determine the NGAL as

well as a preoperative blood collection for the last preoperative laboratory value control and for the collection of whole blood from the donors.

In addition, the recipient undergoes postoperative urine checks for NGAL determination and regular postoperative cystatin C determinations (see above) up to the seventh postoperative day.

The end of the observation period is reached on the day of discharge; no further follow-up is planned.

B.3 Intended procedure, stress for the patient

B.3.1 Visits

Participation in the study as a donor requires two study-related visits - one before and one after the diet - according to the above-mentioned procedure.

During the two visits, the anthropometric data - body weight, body composition and waist circumference - are collected. A study-related blood sample is also taken during the first visit. In addition, the donors are questioned about their dietary adherence the day before the operation.

B.3.2 Issue of food

The food is handed out during the first visit.

B.3.3 Calorie restriction

As expected, the seven-day calorie reduction leads to a subjective feeling of hunger and weight loss of approx. 1 kg. In our study (Cr_KCH), we observed that the one-week diet (corresponding to the diet in this study) did not cause any objective or subjective complaints in the study participants. There were no safety concerns.

B.3.4 Blood/urine sample assay

There will be two study-related blood value checks - the first as part of the first visit before a possible diet and the second on the morning of the day of surgery for all study participants. The other laboratory tests will be taken preoperatively as part of routine clinical preparation where possible.

Post-operative blood samples are taken, where possible, via the central venous catheter (CVC) inserted during the operation and generally do not require an additional puncture.
In addition to the clinical routine, urine samples will be collected from all study participants on the day of surgery and three further post-operative urine samples will be collected exclusively from the recipient.

B.3.5 Taking tissue samples

The removal of punch biopsies from the donor organs and the removal of excess tissue is carried out exclusively as part of regular medical care and clinical routine by the transplant surgery department as part of the explantation or transplantation procedure for histopathological assessment of the donor organ and for safe organ re-implantation
Sufficient material should be collected from 5 patients per group. It is expected that the amount of tissue will not be sufficient for reliable molecular biological testing in every included study participant, so that a higher number of participants up to a maximum of 10 patients per group will be used.
There is no additional exposure due to tissue sampling.

B.4 Patient selection

B.4.1 Selection of test subjects

Patients (male and female) undergoing living donor kidney transplantation are included in the study
Two study arms are formed.

- Donors who are preconditioned by means of calorie restriction
- Donors who are not preconditioned as a control group

The aim is to preserve sufficient excess tissue and blood from 5 patients in each study arm for scientific purposes. According to surgical experience (Prof. Dr. D. Stippel), it can be assumed that 8 patients in each study arm will be operated on for this purpose (we assume that 30% of the patients will not have sufficient excess tissue). As soon as material has been obtained from 5 patients in each group, the study will be terminated. A maximum number of 10 patients in each study arm will not be exceeded.
Assignment to the two groups is initially alternating. Randomization is not possible due to the small number of patients

B.4.2 Inclusion criteria

- Consenting person > 18 years
- Planned living kidney donation
- Written consent in the case of legal capacity
- BMI ≥ 18.5 kg/m²

B.4.3 Exclusion criteria

none

B.4. Case number calculation

No preliminary results are available that would allow the number of cases to be calculated. This study is therefore designed as a proof-of-principle study.

B.5 Type of examination

Monocentric, non-blinded, non-randomized intervention study

B. Legal provisions

Data protection regulations, ICH-GCP

B. Non-authorized medicinal products

Not applicable.

B.8 Complications and risks

The formula diet used is not a medicinal product. Side effects as defined by the AMG/MPG therefore do not occur. Incompatibilities are nausea, vomiting or diarrhea. These are documented and reported to the ethics committee on request. As expected, the seven-day calorie reduction leads to a subjective feeling of hunger and weight loss of approx. 1 kg. These are not side effects.

It should be mentioned that we are currently conducting a study in patients with heart disease using the same dietary regimen, and that there are no relevant safety concerns even shortly

before recruitment is completed. In addition, the study applied for here will largely include healthy subjects (living kidney donors).

B.9 Risk-benefit assessment

Pharmacological renal protection against ischemia-inducing events appears possible in animal models, but all translational approaches in clinical studies have failed, so that there is currently no reliable preventive measure for nephroprotection.

Experimental animal studies have shown that short-term calorie restriction leads to significantly increased resistance to ischemic organ damage. Studies on preoperative diets have not yet been completed (clinicaltrials.gov, NCT01534364, NCT01879839), but a feasibility study conducted by van Ginhoven et al (van Ginhoven, de Bruin et al. Clin Transplant 2011) showed that preoperative calorie restriction is safe and feasible in humans.

The diet used here corresponds to a calorie reduction as it is usually used for weight reduction or in the initial phase of a dietary change (Hauner et al. 2007) .

Due to the potential benefits for patients in the absence of alternative nephroprotective measures prior to surgery, the risks to the patient arising from the preoperative diet are negligible.

B.10 Interim evaluation and termination criteria

An interim evaluation is not planned.

The trial director is authorized to terminate the trial prematurely due to relevant medical or ethical concerns or lack of feasibility of the trial. In such a case, the reasons for the premature termination of the trial are documented in detail. If an investigator has ethical concerns regarding the continuation of the trial, this must be reported immediately to the trial director.

B.11 Content of the patient information / declaration of consent

All study participants (donors and recipients) must give their express written consent to take part in the study before participating. They will be informed about the details of the study verbally and in writing by a medical member of the study team.

(Patient information: see appendix).

B.12 Data protection

All clinically available data as medical history (in particular previous illnesses and diagnostic results) is coded and stored pseudonymously. No patient names are stored. The data is not accessible to third parties. All patient data is subject to medical confidentiality.

B.13 Insurance cover

Subject insurance will be taken out for the participants in this study. Insurance cover will be applied for with the help of this protocol.

C. Abstract

1. rationale / motivation and clinical background of your project proposal

The prevention of expected acute kidney failure, e.. in the context of interventions or operations, is possible using different preconditioning regimens. Short-term pre-interventional calorie restriction in particular has been shown to be very effective in animal experiments. The underlying mechanisms are not understood and have so far only been investigated in animal experiments. It remains unclear to what extent these findings from animal experiments can be transferred to humans. Living kidney donation represents a unique opportunity to obtain and analyze blood and tissue samples of the kidney (as well as the vascular endothelium and fatty tissue) from healthy volunteers without additional risk for the donor and recipient, as a so-called zero biopsy is routinely performed after organ removal.

In contrast to cadaveric kidney transplants, living kidney transplants offer the option of planning and implementing a diet preoperatively. In addition, the donors are by definition healthy individuals, so that calorie restriction poses no health risk. The effect of preoperative calorie restriction on a molecular level (transcriptome, lipidome, metabolome, epigenome, proteome, phosphoproteome) can be investigated by means of a preoperative diet. At the same time, this provides the opportunity to detect a functional effect of calorie restriction on graft function in the recipient.

2 Explicit research question and objective of the study

The aim of this pilot study is to analyze the molecular mechanisms of dietary preconditioning using blood and tissue samples obtained in planned living kidney transplants and to investigate their influence on graft characteristics and function in the recipient after transplantation.

3. **ethical issues**

The study is designed as an interventional proof-of-principle study to further investigate promising approaches from previous animal experiments by means of molecular biological expression profiles in the kidney using human blood and tissue samples. It helps to clarify the extent to which the findings from animal experiments can be transferred to humans. This is still unclear at present.

There is no particular risk to the patient as a result of the above-mentioned examinations. The patient's treatment is not interfered with and care is taken not to disrupt the course of regular medical care. The applicable data protection regulations are complied with.

Cologne, January 29, 2015 PD Dr. med. Volker Burst
(Clinic II for Internal Medicine)

References

1) Ahmet I, Wan R, Mattson MP, Lakatta EG, Talan M. Cardioprotection by intermittent fasting in rats. Circulation 2005; 112: 3115-21.

2) Bein B, Meybohm P. [Organ protection by conditioning]. Anasthesiol Intensivmed Notfallmed Schmerzther 2010; 45: 254-61; quiz 262.

3) Hauner H, Buchholz G, Hamann A, Husemann B, Koletzko B, Liebermeister H, Wabitsch M, Westenhöfer J, Wirth A, Wolfram G (2007). Guideline Prevention and Therapy of Obesity. D. D.-G. German Obesity Society, German and D. G. f. E. Society for Nutrition.

4) Mitchell JR, Verweij M, Brand K, van de Ven M, Goemaere N, van den Engel S, Chu T, Forrer F, Muller C, de Jong M, van IW, JN IJ, Hoeijmakers JH, de Bruin RW. Short-term dietary restriction and fasting precondition against ischemia reperfusion injury in mice. Aging Cell 2010; 9: 40-53.

5) van Ginhoven TM, de Bruin RW, Timmermans M, Mitchell JR, Hoeijmakers JH, Ijzermans JN. Pre-operative dietary restriction is feasible in live-kidney donors. Clin Transplant 2011; 25: 486-94.

6) van Ginhoven TM, Mitchell JR, Verweij M, Hoeijmakers JH, Ijzermans JN, de Bruin RW. The use of preoperative nutritional interventions to protect against hepatic ischemia-reperfusion injury. Liver Transpl 2009; 15: 1183-91.

7) Yu ZF, Mattson MP. Dietary restriction and 2-deoxyglucose administration reduce focal ischemic brain damage and improve behavioral outcome: evidence for a preconditioning mechanism. J Neurosci Res 1999; 57: 830-9.

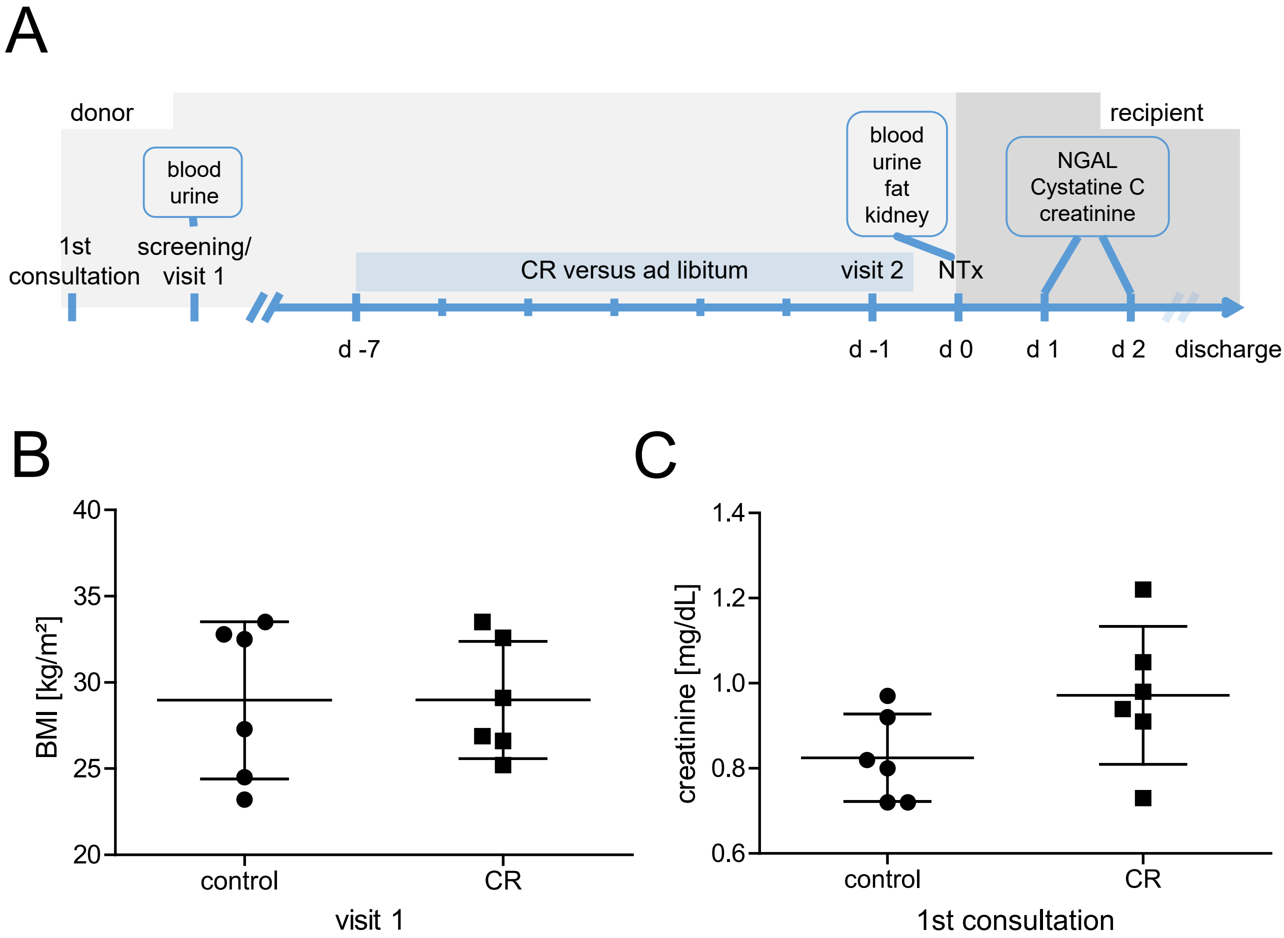

Figure S1: Timeline and baseline characteristics. A. Timeline of the trial procedures. **B.** Body-mass-indices of the control and the CR-group at visit 1. **C.** Creatinine values of the control and the CR-group at the time of the first consultation. **CR:** calorie restriction.

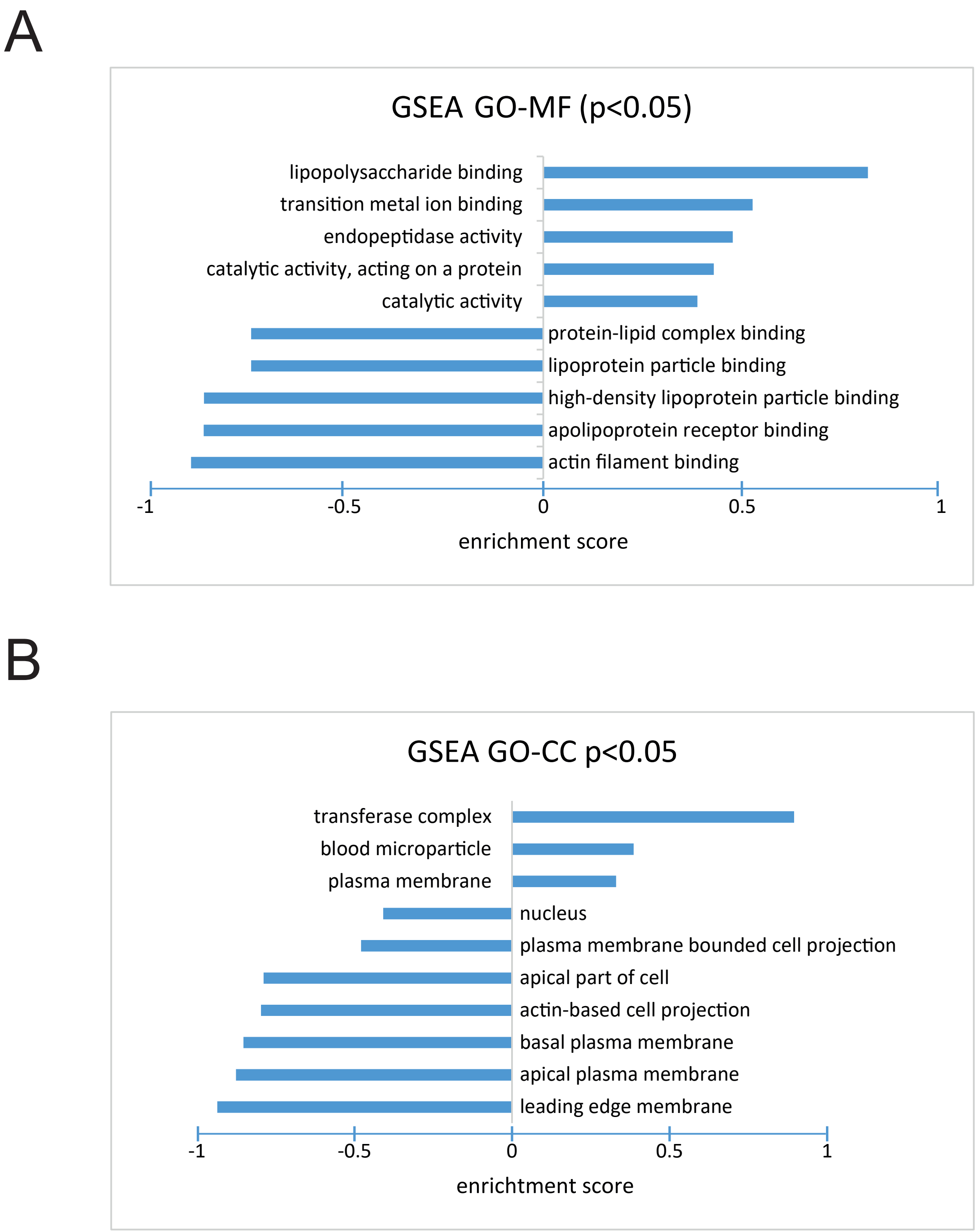

Figure S2: Geneset enrichment analysis (GSEA) of blood serum proteomics. A. GSEA results of GO-MF terms. **B.** GSEA results of GO-CC terms.

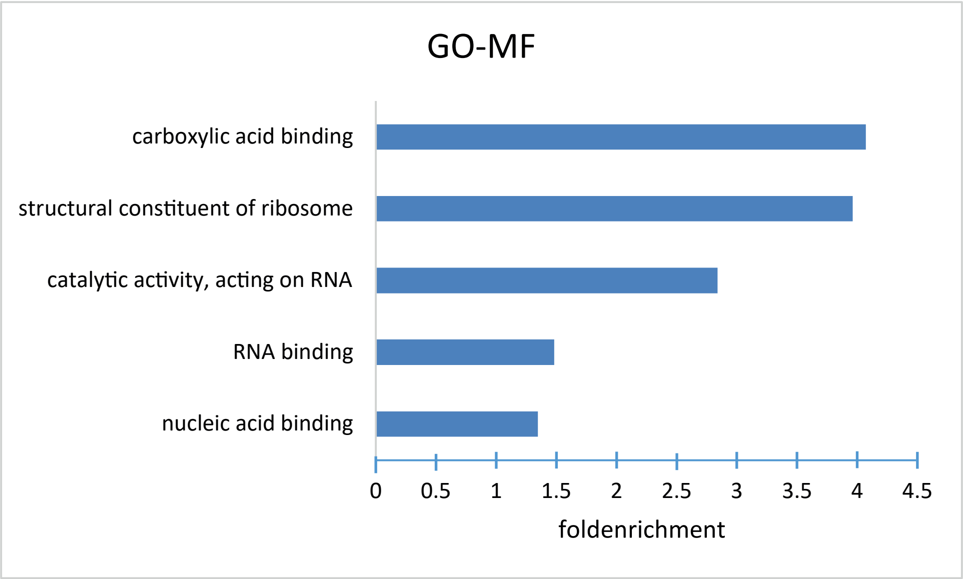

Figure S3: GO-enrichment of GO-MF terms.
